# Tissue localization of collagenase and leucine aminopeptidase in the bovine filarial parasite *Setaria cervi*

**DOI:** 10.1186/1475-2883-5-7

**Published:** 2006-05-22

**Authors:** Daya R Pokharel, Reeta Rai, Pankaj Kumar, C M Chaturvedi, Sushma Rathaur

**Affiliations:** 1 Department of Biochemistry, Faculty of Science, Banaras Hindu University, Varanasi-221005, India; 2 Department of Zoology, Faculty of Science, Banaras Hindu University, Varanasi-221005, India

## Abstract

**Background:**

Like other helminth proteases, filarial proteases have also been shown to require for parasite survival inside the host and mediate various physiologic processes such as tissue invasion, feeding, embryogenesis and host immune evasion. Many of these proteases have shown potential for vaccines and chemotherapeutic agents against active filarial infections. *Setaria cervi *is a bovine filarial parasite and serves as a good parasite model for the studies in lymphatic filariasis. Recently, a 175 kDa collagenase and leucine aminopeptidase (LAP) have been purified and characterized from the bovine filarial parasite *S. cervi *and shown to be potential vaccine candidate and diagnostic marker, respectively for human lymphatic filariasis. However, their tissue localizations and putative roles in the parasite biology have not yet been examined and thus remain unclear. Therefore, the current study attempts to localize and explore the putative roles of these two enzymes in *S. cervi*.

**Methods:**

The tissue distributions of 175 kDa collagenase and leucine aminopeptidase in *S. cervi *were examined by immunohistochemical and histochemical methods, respectively. Immune sera obtained from the jirds immunized with collagenase served as primary antibody, rabbit anti-mouse IgG-HRP conjugate as secondary antibody and DAB as the substrate for the immunostaining of collagenase. Leu-βNA was used as the substrate for the histochemical staining of LAP.

**Results:**

Both the collagenase and LAP were present in the body wall; however, they differ in their distribution pattern in different layers of body wall. Collagenase was mainly localized in epicuticle, cuticle, syncytial hypodermis and the nerve cord region whereas LAP was more concentrated in epicuticle, longitudinal muscle layers and almost absent or very faintly stained in syncytial hypodermis and nerve cord region. Both collagenase and LAP showed their common distributions in intestine, uterus and mature eggs, growing embryos and mf. Very strong immunostaining of collagenase in the outer body surface of the parasite indicates its major role in host-parasite relationship whereas the presence of LAP in muscular region suggests its role in tissue remodeling. The common presences of collagenase and LAP in the *S. cervi *intestine, ovary, uterus, eggs and mf suggest that they also have collaborative roles in molting, nutrition and embryogenesis. The data obtained on their immunological characterizations and their presence in important parasite organs give strong indication that they are critical for the survival of filarial parasite and thus can be good vaccine candidates and/or diagnostic markers for human lymphatic filariasis.

**Conclusion:**

The manuscript reports for the first time the tissue distribution of collagenase and LAP in the bovine filarial parasite *S. cervi *and discuss their putative roles *in vivo*. Our findings also open the avenue to examine the roles of these two proteases *in vivo*, which will require further experiments like using their natural substrates and/or specific inhibitors in each tissues.

## Background

Proteases of various mechanistic classes have been identified in various filarial and other helminth parasites and genes encoding them have been isolated and cloned. These parasite proteases are pivotal for the parasitic existence inside the hostile environment of the hosts. Beside their general protein processing and catabolic functions, proteases have been found to be critical for parasite feeding, host immune evasion, embryogenesis, molting and tissue invasion [1-6]. They have been shown to be highly immunogenic and thus exploited as serodiagnostic markers and vaccine candidates for many helminth infections [7-9]. Compared to their host counterparts; parasite proteases have distinct structural and biochemical properties and cellular locations. This disparate nature of parasite proteases has also opened opportunities for chemotherapy against many parasitic diseases [10-12]. Thus exploring biochemical and immunological properties, structures and roles of parasite proteases *in vivo *is an essential step towards the identification and development of ideal diagnostic markers and, vaccine and drug targets for the control of parasitic diseases.

*Setaria cervi *is a filarial parasite of Indian buffaloes and resembles with *Wuchereria bancrofti *in its nocturnal periodicity and antigenic pattern. Being a bovine parasite, its use as the parasite model is not restricted by the inherent ethical and practical limitations associated with humans and therefore serve as a good model parasite for the studies in lymphatic filariasis. Recently, two metalloproteases: a 175 kDa collagenase and leucine aminopeptidase (LAP), have been purified and characterized from adult female *S. cervi *in authors' laboratory. The *S. cervi *collagenase was shown to have important roles in host immune evasion and immunoprotection. This enzyme specifically cleaved human IgG in vitro and was inhibited by the antibodies raised against it in jirds. The enzyme also showed a very high cross-reactivity with putatively immune human sera collected from filarial endemic zones [13,14] and showed antigenic similarity to *Brugia malayi *[Unpublished observation]. Both *in vitro *antibody dependent cell mediated cytotoxicity (ADCC) assay [15] and vaccine trial carried out in jirds against *B. malayi *using purified collagenase suggested it to be an effective vaccine candidate against human lymphatic filariasis [Unpublished observation].

The *S. cervi *LAP has been characterized as a member of M17 family of Zn-containing metalloprotease. This enzyme was inhibited by Zn-chelating inhibitor 1, 10-phenanthroline and other metalloprotease inhibitors like EDTA, amastatin and bestatin, and showed very high preference towards the substrate leucine para-nitroannilide (Leu-pNA). The enzyme purified from adult worm soluble extract showed high specificity with microfilaraemic sera from individuals infected with *W. bancrofti *[Unpublished observation]. The later observation has indicated its potential use as a diagnostic marker for filarial infection. A vaccine trial with LAP is currently under progress and results are awaited. Vaccinations with aminopeptidase alone or in combination with other proteases have yielded very high protection against *Fasciola hepatica *and *Haemonchus contortus *infections in sheep [9,16,17]. Thus, it may be anticipated that *S. cervi *LAP will too provide sufficient host protection against *B. malayi *infection in jirds.

Although biochemical and immunological properties of *S. cervi *collagenase and LAP have been studied in detail, their localizations and possible physiological roles inside the parasite body have not yet been examined and thus remain unclear. Therefore, to address the potential in vivo roles of collagenase and LAP, it would be helpful to establish their precise locations in the parasite tissues. Reports on tissue localization of collagenase in filarial and other helminth parasites are scanty. However, tissue localization of related metallo-, cysteine and acidic proteases have been carried out in various filarial [1,3] and non-filarial parasites [18-20] and have shown their presence mainly on intestinal microvilli, cuticle, hypodermis, ovary, uteri, and periphery of eggshell and growing embryos. On the other hand, LAP has been detected, purified and characterized in many other related parasites e.g. in *F. hepatica *[21], *Ascaris suum *[22-24], *Schistosoma mansoni *and *S. japonicum *[25,26]. In case of *F. hepatica*, the enzyme was localized to the cells of the gut epithelium of adult flukes [21]. Immunolocalisation studies in *S. mansoni *showed that LAP was localized in the gastrodermal cells surrounding the gut lumen and surface tegument and eggs [25,26].

In this study, we therefore investigated the localization of collagenase and LAP in the adult female *S. cervi *tissues including eggs and mf still present within the worm ovary and uterus by IHC and histochemical staining respectively and discuss their putative physiological roles in the parasite body.

## Methods

### Filarial parasites and tissue preparation

Adult, motile *S. cervi *worms were procured from the peritoneal folds of freshly slaughtered Indian buffaloes. They were brought to the laboratory from local abattoir in Kreb's Ringer solution supplemented with streptomycin (100 μg/ml), penicillin (100 μg/ml), glutamine (2 mM) and glucose (1%). Worms were washed six times with PBS, divided into two groups and chopped into short fragments. The parasite tissues aimed for IHC of collagenase were fixed in Bouin's fixer for 24 h at room temperature and embedded in paraffin block until use. The next group of chopped parasites aimed for histochemistry of LAP was fixed in formol-calcium at 4°C for 24 h and embedded in paraffin block until use.

### Immunohistochemistry (IHC) of collagenase

Thin sections (5 μm) of the paraffin embedded tissues of *S. cervi *aimed for IHC of collagenase were cut using a microtome and spread over 1% gelatin coated slides which were covered with distilled water and warmed at 55–60°C. The immunohistochemistry was performed as described elsewhere [27]. Briefly, sections were deparaffinised with three changes of xylene and rehydrated through graded alcohols and finally with water. The endogenous peroxidase activity was inactivated by incubating these sections in 3% H_2_O_2 _in methanol for 30 min at room temperature. Next, the slides were rinsed with three changes of PBS and the sections were incubated with 2% normal rabbit blocking serum for 1 h. The excess blocking serum was shed off and the sections were further incubated overnight at 4°C with primary antibody obtained from jirds (*Meriones unguilatus*) immunized with purified collagenase (1:200 dilutions). The sections were rinsed thrice with PBS and incubated for 1 h with horseradish peroxidase conjugated secondary antibody (1:2500 dilutions) at room temperature. The antigen-antibody complex was then visualized by incubating the sections with 3, 3'-diaminobenzidine (DAB) solution (1 mM DAB, 50 mM Tris-HCl, pH 7.6, and 0.015% H_2_O_2_) in dark for 15–30 min. The slides were then counterstained with Meyer's hematoxylin and treated through graded alcohols and xylene, and mounted with DPX. Sections exposed to preimmune jirds sera were always included as negative controls. Permanent slides were examined using a light microscope (Nikon Eclipse E800, Nikon, Tokyo, Japan) and photographed with Nikon digital Camera DXM 1200.

### Histochemical staining of leucine aminopeptidase (LAP)

The parasite tissues fixed in formol-calcium were cut into thin sections (5 μm) and spread over gelatin coated slides as described above for collagenase. The sections were deparaffinised with xylene and rehydrated through graded alcohols and water. LAP histochemical staining was performed according to the method described elsewhere [28]. Briefly, the rehydrated sections were incubated with the incubating solution [L-Leu-β-naphthylamide 0.5 mM, Potassium cyanide (0.00065%), sodium chloride (0.34%) and Fast Blue B salt (0.05%)] in a moist dark chamber for 2 h. Next, the slides were rinsed consecutively in 0.85% saline, 0.1 M copper sulphate and again with saline for 2 min each. The sections were then counterstained with 2% methyl green for 3 min and rinsed in distilled water. Finally the counterstained sections were treated through the graded alcohols, xylene and mounted in DPX for permanent slide preparation. Sections for negative controls were incubated in the incubating medium without the substrate. The slides were observed under a light microscope (Nikon Eclipse E800, Nikon, Tokyo, Japan) and photographed with Nikon digital Camera DXM 1200.

## Results

### Distribution of collagenase in *S. cervi*

We examined the localization of 175kDa collagenase in various tissues of adult *S. cervi *worms including eggs and microfilariae using IHC and assessed the immunostaining from at least five sections obtained from different body regions of the three separate worms. The results of immunostaining are summarized in Table 1.

**Table I:** Tissue Distribution of Collagenase and LAP in *S. cervi*^a^.

	**Relative Staining Intensity**	
	
**Tissues**	**Collagenase**	**Leucine Aminopeptidase (LAP)**
**Epiuticle**	++++	++++
**Cuticle**	++++	-/+
**Syncytial hypodermis**	++++	+
**Longitudinal muscle layers**	++	++++
**Endodermis**	+++	++++
**Intestine**	+++	++++
**Ovary growth zone**	++	**NS**
**Ovary germinal zone**	++	++
**Uterine wall**	+++	++++
**Eggs**		
**(a) Immature**	-	++
**(b) Mature**	+++	+++
**Embryo**	+++	+++
**Microfilariae (mf)**	++	++++

^a^++++: Strongly positive; +++: Positive; +++: Moderately positive; ++: Positive; + or +/-: Faintly positive: -: Negative; NS: Not Studied

**Figure 1:**
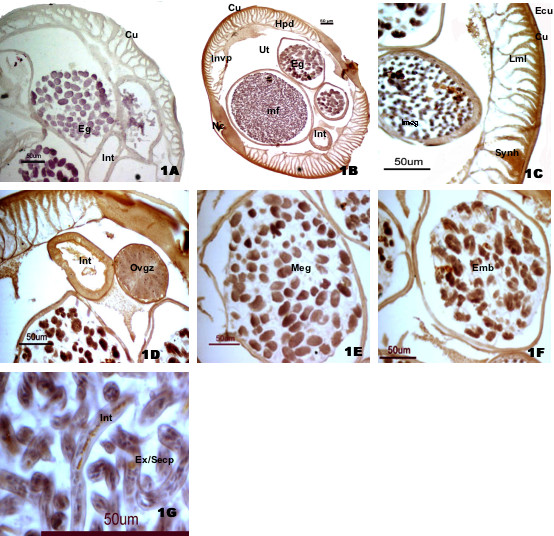
Immunohistochemical staining of *S. cervi *175 kDa Collagenase. (1A) Control Section, (1B) Transverse section showing positive staining for 175 kDa collagenase; (1C) Body wall; (1D) Intestine and ovary growing zone; (1E) Uterus showing mature egg; (1F) Growing embryos; (1G) Microfilaria showing immunostaining in intestine and excretory-secretory pore.

The appearance of dark brown color of DAB was considered as the basis for evaluation of positive staining in the parasite tissues. Very strong immunostaining was observed in the epicuticle and cuticle followed by syncytial hypodermis. Good staining was observed in longitudinal muscle layers, endodermis, intestinal wall, uterine wall, ovary (growth and germinal zones), and periphery of mature eggs and growing embryo inside the uterus. The intensity of enzyme staining was found to be gradually increased from immature ovary to fully-grown ovary. The staining in mf was, however, found to be localized in certain secretary pores and body cavity when viewed under higher magnification (Fig 1B–G). Faint staining was observed in the innervation process beneath the body wall. No staining was observed in the spaces between longitudinal muscle layers and the tissue inside the uterus. No such staining was observed in control sections in which preimmune jirds sera were used as primary antibody (Fig 1A).

### Distribution of LAP in *S. cervi*

**Figure 2:**
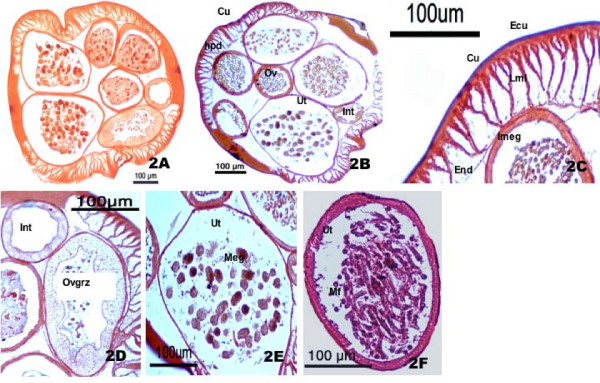
Histochemical staining of *S. cervi *Leucine aminopeptidase. (2A) Control Section, (2B) Transverse section showing positive staining for LAP; (2C) Body wall; (2D) Intestine and ovary germinal zone (2E) Uterus containing mature eggs; (2F) Microfilaria inside uterus showing positive staining for LAP.

Next, we examined the distribution of LAP in adult worm tissues including eggs, developing embryos and mf by histochemical staining using L-Leu-β-Naphthylamide as substrate. The distribution pattern and staining intensity are presented in Table 1. The pink to magenta colour obtained due to reaction of β-naphtylamine and Fast Blue B salt in the presence of Cu ^2+ ^ions against reddish brown background was considered as the positive staining. Strong staining was observed in epicuticle, longitudinal muscle layers, uterine walls and mf surface followed by intestine and endodermis. The young ovary walls, periphery of eggs and developing embryos showed moderate staining. No enzyme staining was observed in and around the nerve cord, syncytial hypodermis and tissue inside the uterus (Fig. 2A–F). Here again the intensity of enzyme staining was found to increase with the maturity of ovary and eggs. The staining intensity of LAP in mf surface was far higher compared to immunostaining of collagenase.

## Discussion

We have investigated the tissue localizations of two important proteolytic enzymes viz. 175kDa collagenase and leucine aminopeptidase in the bovine filarial parasite *S. cervi *by immunohistochemical and histochemical staining, respectively.

While comparing the distribution patterns of collagenase and leucine aminopeptidase in *S. cervi *we observed a marked difference in their distribution pattern in body wall and mf surface (Fig 1 and 2). Whereas the collagenase was highly expressed in epicuticle and cuticle region and syncytial hypodermis (Fig 1C), expression of LAP was more prominent in epicuticle, longitudinal muscle layers, endodermis and intestine (Fig 2C). Unlike collagenase, the staining for LAP was not very strong in epicuticle and almost no or faint staining was observed in cuticle, nerve cord and syncytial hypodermis regions (Fig 2B). Furthermore, only body cavity and certain surface bound excretory/secretory pores were positively stained for collagenase, staining for LAP was widespread and stronger in mf surface (Fig 1G and 2F). This difference in enzyme distribution pattern in adult worm's body wall and mf surface indicates their different job priority in these regions. However, almost similar distribution patterns for both the enzymes were noted in intestine, mature eggs, embryos and uterine wall thus indicating their parallel or collaborative functions at least in these regions. The developing ovary wall and eggs were either negative or faintly stained whereas uterine wall, mature eggs and growing embryos showed good staining for both the enzymes (Fig 1 and 2).

The cuticle in all nematode parasites is an extracellular hydroskeleton that is relatively inert, structurally robust, and selectively permeable [29]. Molting is a multistage process, involving breakdown of the connections between the cytoskeleton of the hypodermis and the old cuticle (apolysis), elaboration of the new cuticle, and shedding of the old cuticle (ecdysis) [30]. Both apolysis and ecdysis require the involvement of proteases for the degradation of cuticular proteins. Additionally, proteases may also be involved in the processing of pro-proteins that are subsequently incorporated into the new cuticle [29]. Several type of proteases such as serine, cysteine and/or metalloproteases that are active during molting have been described in filariae [31-33] and other helminth parasites such as *Phocanema decipiens *[34], *Ancylostoma sps*. [35], *Haemonchus contortus *[18,36-38] and *C. elegans *[2,39].

In filarial nematodes, the molt from third-stage larvae (L3) to fourth-stage larvae (L4) begins immediately after the nematodes enter a vertebrate host and require several types of proteases. Irreversible cysteine protease inhibitors delay or stop this molting process *in vitro *in case of L3 larval stages of *Onchocerca volvulus *[32], *Dirofilaria immitis *[40] and *B. pahangi *[1]. In these nematodes, the L3 larvae were found to be viable, but were unable to exsheath. Labeled cysteine protease inhibitors or antibodies located the cathepsin L-like protease activity to the fluid-filled space between the nematode tegument and the exsheathing cuticle. More recently, cathepsins L (CPL)-like genes were cloned from *O. volvulus *(*Ov*-*cpl*-*1*) and *C. elegans *(*Ce*-*cpl*-*1*) and shown to localize in the cuticles of molting worms, thus implicating they also have a potential role in the molting process [2]. A conserved metalloprotease, NAS 37, has also been shown to involve in the final stage of molting in *C. elegans *[39]. This protease is expressed in hypodermal cells four hour before ecdysis during all larval stages and is shed in the cuticle after ecdysis. An ortholog of NAS-37 has been reported from other parasitic nematodes, such as *H. contortus *which undergo a similar shedding process [36].

Although, viability studies of *S. cervi *are yet to be done using specific inhibitors of collagenase and LAP, their presence in outer surface of mf definitely indicates their roles in larval molting. As the staining intensity of LAP was higher and widespread on mf surface, this enzyme should primarily be involved for molting process. Collagenase, in addition to its possible role in larval molting, must be involved in immune evasion and migration in the host tissues. It has already been shown that the *S. cervi *collagenase (both secreted and somatic) can cleave human IgG molecules precisely at the papain cleavage site of hinge region and hydrolyze connective tissue proteins like collagen *in vitro*. The enzyme was also shown to prevent the antibody-mediated attachment of eosinophils to the parasite surface when tested in jirds by *in vitro *ADCC assay [15]. However, their expression in adult stage indicates that these enzymes might have other functions too. The high expression of LAP to the adult longitudinal muscle layers compared to collagenase suggests that it also plays a major role in tissue remodeling in these locations.

The hatching of parasite eggs represents a critical phase in a parasite's lifecycle. In general, the hatching process is initiated by an appropriate signal, which may be provided by the host or environment. These environmental signals may stimulate the embryo to secrete compounds, for example, hatching enzymes [41,42], which then degrade the eggshell or cyst and thus facilitate hatching. Hatching enzymes have been identified in *Ascaris spp*. [43,44]*H. contortus *[45] and *Heligmosomoides polygyrus *[46] and include molecules such as proteases, lipases, chitinases, α- and β-glycosidases and leucine aminopeptidases. Unlike other helminth parasites, filarial worms such as *S. cervi *are ovoviviparous and thus directly release mf in host blood circulation. The presence of both collagenase and LAP on the egg and embryo surface clearly indicates their roles in eggshell remodeling, embryogenesis and release of mf from uterus. An interesting observation noted was that the expressions of both enzymes gradually increased as the eggs maturation progress indicating their roles in hatching and embryogenesis. Furthermore, an inverse relationship was observed between thickness of ovary wall and expression of both the enzymes. This unique observation implies that both LAP and collagenase are intimately involved in releasing of mf from uterus.

The involvement of proteases in the egg hatch of *S. cervi *provides the opportunity to investigate novel means for controlling the egg hatch process. Further characterization of these two proteases in terms of their tertiary structure may enable the design of specific inhibitors in order to block the egg hatch process. The use of specific protease inhibitors which are non-toxic to the host, but which display specificity for the target protease represents a novel approaches to chemotherapy. Such an approach has been investigated in *Schistosoma spp*. where specific inhibitors are being designed to block the activity of an elastase protease in order to prevent skin penetration of this parasite [47,48].

Like other hematophagus helminths, *S. cervi *parasites also require blood and other proteins as a source of nutrients to mature and reproduce. The expression of both the proteases in the gastrodermis clearly supports the view that they are intimately involved in digestion of protein food of the parasite. In a typical proteolytic cascade that ensues during protein metabolism in helminth parasites, collagenase being an endopeptidase can be expected to play a role in cleaving of large polypeptide chains before LAP release free amino acids from the partially digested proteins at the final stage of cascade. Since LAP is almost non-functional below pH 5.0, it might be active within the gastrodermal cells and help in activation and secretion of other proenzymes of endo- and exopeptidases that are active at low pH and also release free amino acids from absorbed peptide fragments.

In the present study, we have for the first time established the tissue localization of collagenase and LAP in adult female *S. cervi *including eggs and mf and discussed their putative roles for the survival and persistence of parasite within the host. Our findings also open the avenue to examine the roles of these two proteases *in vivo*, which will require further experiments like using their natural substrates and/or specific inhibitors in each tissues.

## Authors' contributions

This paper is the part of PhD research of Mr Daya Ram Pokharel. Hence, Daya Ram Pokharel had the main responsibility for the study (planning, data analysis, and writing). Rita Rai was involved in procuring adult *S. cervi *worms and revision of the manuscript. Pankaj Kumar was involved himself and helped in the conduction of experiments. Chandra M Chaturvedi provided the laboratory facility and supervised the experimental part. Sushma Rathaur is PhD supervisor and helped to plan the study, analyze results, and write the manuscript.

## References

[B1] Guiliano DB, Hong X, McKerrow JH, Blaxter ML, Oksov Y, Liu J, Ghedin E, Lustigman S (2004). A gene family of cathepsin L-like proteases of filarial nematodes are associated with larval molting and cuticle and eggshell remodeling. Mol Biochem Parasitol.

[B2] Hashmi S, Zhang J, Oksov Y, Lustigman S (2004). The *Caenorhabditis elegans *cathepsin Z-like cysteine protease, Ce-CPZ-1, has a multifunctional role during the worms' development. J Biol Chem.

[B3] Lustigman S, Zhang J, Liu J, Oksov Y, Hashmi S (2004). RNA interference targeting cathepsin L and Z-like cysteine proteases of *Onchocerca volvulus *confirmed their essential function during L3 molting. Mol Biochem Parasitol.

[B4] Sajid M, McKerrow JH (2002). Cysteine proteases of parasitic organisms. Mol Biochem Parasitol.

[B5] Tort J, Brindley PJ, Knox D, Wolfe KH, Dalton JP (1999). Proteinases and associated genes of parasitic helminths. Adv Parasitol.

[B6] Williamson AL, Brindley PJ, Knox DP, Hotez PJ, Loukas A (2003). Digestive proteases of blood-feeding nematodes. Trends Parasitol.

[B7] Cordova M, Reategui L, Espinoza JR (1999). Immunodiagnosis of human fasciolosis with *Fasciola hepatica *cysteine proteinases. Trans Royal Soc of Trop Med and Hyg.

[B8] Rokni MB, Massoud J, O'Neill SM, Parkinson M, Dalton JP (2002). Diagnosis of human fasciolosis in the Gilan province of Northern Iran: application of cathepsin L-ELISA. Diag Microbiol Infect Dis.

[B9] Piacenza L, Acosta D, Basmadjian I, Dalton JP, Carmona C (1999). Vaccination with cathepsin L proteinases and with leucine aminopeptidase induces high levels of protection against fasciolosis in sheep. Infect Immun.

[B10] Coombs GH, Mottram JC (1997). Parasite proteinases and amino acid metabolism: possibilities for chemotherapeutic exploitation. Parasitol.

[B11] McKerrow JH, Engel JC, Caffrey CR (1999). Cysteine protease inhibitors as chemotherapy for parasitic infections. Bioorg Med Chem.

[B12] Selzer PM, Pingel S, Hsieh I, Ugele B, Chan VJ, Engel JC, Bogyo M, Russell DG, Sakanari JA, McKerrow JH (1999). Cysteine protease inhibitors as chemotherapy: lessons from a parasite target. Proc Natl Acad Sci USA.

[B13] Singh RN, Rathaur S (2003). *Setaria cervi *: *In vitro *released collagenases and their inhibition by *Wuchereria bancrofti *infected sera. J Helminth.

[B14] Srivastava Y, Gupta S, Pokharel DR, Rathaur S (2004). *Setaria cervi *collagenase: IgG cleavage and inhibition by *W. bancrofti *infected sera. Acta Parasitol.

[B15] Srivastava Y, Bhandari YP, Reddy MVR, Harinath BC, Rathaur S (2004). An adult 175 kDa collagenase antigen of *Setaria cervi *in immunoprophylaxis against *Brugia malayi*. J Helminth.

[B16] Andrews SJ, Rolph TP, Munn EA, Taylor MA (1997). Duration of protective immunity against ovine haemonchosis following vaccination with the nematode gut membrane antigen H11. Res Vet Sci.

[B17] Newton SE, Munn EA (1999). The development of vaccines against gastrointestinal nematode parasites, particularly *Haemonchus contortus*. Parasitol Today.

[B18] Shompole S, Jasmer DP (2001). Cathepsin B-like cysteine proteases confer intestinal cysteine protease activity in *Haemonchus contortus*. J Biol Chem.

[B19] Williamson AL, Brindley PJ, Abbenante G, Prociv P, Berry C, Girdwood K, Pritchard DI, Fairlie DP, Hotez PJ, Dalton JP, Loukas A (2002). Cleavage of hemoglobin by hookworm cathepsin D aspartic proteases and its contribution to host-specificity. FASEB J.

[B20] Williamson AL, Lecchi P, Turk BE, Choe Y, Hotez PJ, McKerrow JH, Cantley LC, Sajid M, Craik CS, Loukas A (2004). A Multi-enzyme cascade of hemoglobin proteolysis in the intestine of blood-feeding hookworms. J Biol Chem.

[B21] Acosta D, Goni F, Carmona C (1998). Characterization and partial purification of a leucine aminopeptidase from *Fasciola hepatica*. J Parasitol.

[B22] Rhoads ML, Fetterer RH (1998). Purification and characterisation of a secreted aminopeptidase from adult *Ascaris suum*. Int J Parasitol.

[B23] Rhodes MB, Marsh CL, Ferguson DL (1969). Studies in helminth enzymology. VI. Aminopeptidases from uterine extracts of *Ascaris suum*. Exp Parasitol.

[B24] Rhodes MB, Marsh CL, Ferguson DL (1969). *Ascaris suum *: Purification and characterization of an intestinal aminopeptidase. Exp Parasitol.

[B25] Abouel-Nour MF, Lotfy M, El-Kady I, El-Shahat M, Doughty BL (2005). Localization of leucine aminopeptidase in the *Schistosoma mansoni *eggs and in liver tissue from infected mice. J Egypt Soc Parasitol.

[B26] McCarthy E, Stacka C, Donnelly SM, Doyle S, Mann VH, Brindley PJ, Stewart M, Day TA, Maule AG, Dalton JP (2004). Leucine aminopeptidase of the human blood flukes, *Schistosoma mansoni *and *Schistosoma japonicum*. Int J Parasitol.

[B27] Cuello AC (1993). Immunohistochemistry II.

[B28] Nachlas MM, Crawford DT, Seligmann AM (1957). Histochemical demonstration of leucine aminopeptidase. J Histochem Cytochem.

[B29] Page AP, Kennedy MW, Harnett W (2001). The nematode cuticle: synthesis, modification and mutants. Parasitic nematodes molecular biology biochemistry and immunology.

[B30] Lee DL, Lee DL (2002). Cuticle, moulting and exsheathment. The biology of nematodes.

[B31] Hong X, Bouvier J, Wong MM, Yamagata GY, McKerrow JH (1993). *Brugia pahangi *: identification and characterization of an aminopeptidase associated with larval molting. Exp Parasitol.

[B32] Lustigman S, McKerrow JH, Shah K, Lui J, Huima T, Hough M, Brotman B (1996). Cloning of a cysteine protease required for the molting of *Onchocerca volvulus *third stage larvae. J Biol Chem.

[B33] Lustigman S (1993). Molting, enzymes and new targets for chemotherapy of *Onchocerca volvulus*. Parasitol Today.

[B34] Davey KG, Kan SP (1968). Molting in a parasitic nematode, *Phocanema decipiens*. IV. Ecdysis and its control. Can J Zool.

[B35] Hotez P, Haggerty J, Hawdon J, Milstone L, Gamble HR, Schad G, Richard F (1990). Metalloproteases of infective *Ancylostoma *hookworm larvae and their possible functions in tissue invasion and ecdysis. Infect Immun.

[B36] Gamble HR, Purcell JP, Fetterer RH (1989). Purification of a 44 kilodalton protease which mediates the ecdysis of infective *Haemonchus contortus *larvae. Mol Biochem Parasitol.

[B37] Redmond DL, Knox DP, Newlands G, Smith WD (1997). Molecular cloning and characterisation of a developmentally regulated putative metallopeptidase present in a host protective extract of *Haemonchus contortus*. Mol Biochem Parasitol.

[B38] Rogers WP (1965). The role of leucine aminopeptidase in the molting of nematode parasites. Comp Biochem Physiol.

[B39] Davis MW, Birnie AJ, Chan AC, Page AP, Jorgensen AM (2004). A conserved metalloprotease mediates ecdysis in *Caenorhabditis elegans*. Development.

[B40] Richer JK, Hunt WG, Sakanari JA, Grieve RB (1993). *Dirofilaria immitis *: effect of fluoromethyl ketone cysteine protease inhibitors on the third- to fourth-stage molt. Exp Parasitol.

[B41] Sommerville RI, Rogers WP (1987). The nature and action of host signals. Adv Parasitol.

[B42] Rogers WP, Soulsby EJL (1966). Exsheathment and hatching mechanisms in helminths. Biology of parasites.

[B43] Hinck LW, Ivey MH (1976). Proteinase activity in *Ascaris suum *eggs, hatching fluid, and excretions-secretions. J Parasitol.

[B44] Rogers WP (1958). Physiology of the hatching of eggs of *Ascaris lumbricoides*. Nature.

[B45] Rogers WP, Brooks F (1997). The mechanism of hatching of eggs of *Haemonchus contortus*. Int J Parasitol.

[B46] Arnold K, Brydon LJ, Chappell LH, Gooday GW (1993). Chitinolytic activities in *Heligmosomoides polygyrus *and their role in egg hatching. Mol Biochem Parasitol.

[B47] Cohen FE, Gregoret LM, Amari P, Aldape K, Railey J, McKerrow JH (1991). Arresting tissue invasion of a parasite by protease inhibitors chosen with the aid of computer modeling. Biochem.

[B48] Ring CS, Sun E, McKerrow JH, Lee JK, Rosenthal PJ, Kuntz ID, Cohen FE (1993). Structure-based inhibitor design by using protein models for the development of antiparasitic agents. Proc Nat Acad Sci USA.

